# A new perspective on the role of the CREB family of transcription factors in memory consolidation via adult hippocampal neurogenesis

**DOI:** 10.3389/fnmol.2015.00046

**Published:** 2015-08-26

**Authors:** Sylvia Ortega-Martínez

**Affiliations:** Turku Centre for Biotechnology, Åbo Akademi University and University of TurkuTurku, Finland

**Keywords:** adults, mammals, cyclic AMP-responsive element-binding protein, hippocampus, memory, neurogenesis, neuronal plasticity, transcription factors

## Abstract

Adult neurogenesis is the process by which new neurons are generated in the brains of adults. Since its discovery 50 years ago, adult neurogenesis has been widely studied in the mammalian brain and has provided a new perspective on the pathophysiology of many psychiatric and neurodegenerative disorders, some of which affect memory. In this regard, adult hippocampal neurogenesis (AHN), which occurs in the subgranular zone (SGZ) of the dentate gyrus (DG), has been suggested to play a role in the formation and consolidation of new memories. This process involves many transcription factors, of which cyclic AMP (cAMP)-responsive element-binding protein (CREB) is a well-documented one. In the developing brain, CREB regulates crucial cell stages (e.g., proliferation, differentiation, and survival), and in the adult brain, it participates in neuronal plasticity, learning, and memory. In addition, new evidence supports the hypothesis that CREB may also participate in learning and memory through its involvement in AHN. This review examines the CREB family of transcription factors, including the different members and known signaling pathways. It highlights the role of CREB as a modulator of AHN, which could underlie its function in memory consolidation mechanisms.

## The CREB Family of Transcription Factors

Cyclic AMP (cAMP)-responsive element-binding protein (CREB) belongs to the family of leucine zipper transcription factors, which are expressed in a variety of tissues (Yamashima, 2012). In 1986, Marc Montminy and R.H. Goodman first defined cAMP response element (CRE) as a conserved DNA sequence in promoter elements that were activated by cAMP. One or several copies of CRE are present within the regulatory regions of genes. Binding of different transcription factors to CRE regulates RNA polymerase activity, thereby controlling gene expression. A year after CREB was defined, Montminy and Bilezikjian (1987) described CREB as a cellular transcription factor that binds CRE and induces transcription of the somatostatin gene.

The CREB family of transcription factors is now known to include CREB, CRE modulator (CREM), and activating transcription factor-1 (ATF-1). This family belongs to the superfamily of basic region/leucine zipper (bZIP) transcriptional regulators, and all its members mediate cAMP-responsive transcription. CREB and ATF-1 are expressed ubiquitously, whereas CREM is mainly expressed in the neuroendocrine system. Each transcription factor is encoded by a single gene and contains the highly conserved leucine zipper sequence with an adjacent basic region that binds to a CRE DNA sequence. Members of the CREB family also have a regulatory central kinase-inducible domain (KID), although the Q1 and Q2 glutamine-rich regions adjacent to KID are less conserved among different family members (see review Barco et al., 2003).

*Creb1* is the gene responsible for transcription of all known CREB isoforms; the α isoform is the most common, but the β and Δ isoforms are also transcribed. Several approaches have been used to clarify the roles of different CREB isoforms. One study assessed CRE-DNA binding in nuclear extracts obtained from several brain regions of wild-type and CREBΔ/α mutant mice. The results suggested that CRE-DNA–binding complexes contain both CREB and CREM proteins. However, CRE-DNA binding was abolished in the cortex, hippocampus, cerebellum, and amygdala of CREBΔ/α mutant mice. Because these transgenic animals lack CREBΔ and α isoforms, the expression of other forms of CREB, such as CREB-β and CREM, is upregulated. The findings indicate that CREM binding to CRE sites requires the presence of CREBΔ/α, and that CREB-β may not efficiently bind to CRE sites (Pandey et al., 2000). In addition, mice lacking the α and Δ CREB proteins have abnormal long-term, but not short-term memory (STM), indicating that these isoforms are essential for the role of CREB in long-term memory (LTM; Bourtchuladze et al., 1994). In contrast to previous classical studies in adult mice, some studies have focused on the effect of these isoforms on neocortical plasticity in young mice to highlight the age-dependent role of CREB in neuroplasticity. In one of these studies, the CREBα/Δ mutation did not affect plasticity in cortical layers II/III of the younger adolescent mice (1–2 months) indicating that different plasticity processes occur at this age. In the same model, CREBβ expression was upregulated in the barrel cortex of CREBα/Δ knock-out animals, suggesting that this subunit may partly compensate for the loss of the α/Δ isoforms in the young mice. Overall, the study results suggested that CREB isoforms play a role in experience-dependent plasticity in the adult neocortex (Glazewski et al., 1999).

The *Creb1* gene has a complex structure with multiple exons and introns that result in several alternatively spliced mRNAs encoding proteins with distinct transcription-activating or transcription-repressing properties. However, two structural features are present in most members of the CREB family: (a) the well-conserved bZIP domain at the C-terminus, which facilitates dimerization between different family members and participates in the recognition of and binding to CRE sites; and (b) KID (mentioned above), which encloses sites for phosphorylation by protein kinase A (PKA) and other kinases. KID is delineated by two glutamine-rich domains (Q1 and Q2), which are responsible for basal transactivation activity. Q2 connects with TAF_II_ 130 (TBP [TATA-binding protein]-associated factor_II_ 130) and recruits the transcription machinery to the promoter region. Constitutive (Q2) and inducible (KID) domains work together in response to different stimuli that trigger CREB-dependent gene expression (see review Barco et al., 2003). Transcription factors of the CREB family exert their actions as homodimers or heterodimers, but the CREB-CREB homodimer is the most potent transcriptional activator (Dworkin and Mantamadiotis, 2010). CREB isoforms are widely distributed in the adult mouse brain under homeostatic conditions and remain inactive when bound to CRE elements within target gene promoters (Nichols et al., 1992).

The transcriptional activity of CREB depends on its phosphorylation status, which is determined by the opposing actions of protein kinases and phosphatases. Phosphorylation is a key mechanism in signaling and has been described to regulate several processes such as the cell cycle (Liu et al., 2014), cell death (Martin, 2010), DNA damage (Abreu et al., 2013) and neurogenesis (Faigle and Song, 2013). For example, increase in intracellular calcium (Ca^2+^) levels through voltage- and ligand-dependent channels results in increased cAMP levels via activation of G-protein-coupled receptors. Growth factors can also activate receptor tyrosine kinases to augment cAMP levels. All these pathways affect CREB phosphorylation levels. Downstream of neuronal activity, the PKA, mitogen-activated protein kinase (RSK [p90 ribosomal protein S6 kinase]/MAPK), and CaMKIV (Ca^2+^/calmodulin-dependent protein kinase IV) kinase pathways result in CREB phosphorylation at Ser133. PP-1 (protein phosphatase-1) and PP2-A are the major CREB phosphatases, and Ca^2+^-dependent signals can induce intracellular events that lead to phosphorylation or dephosphorylation. The effect of stimulation depends on the nature of the stimulus and its cellular context. For example, activation of *N*-methyl-D-aspartate (NMDA) receptors leads to CREB dephosphorylation in extrasynaptic neurons, while in synaptic sites, it leads to CREB phosphorylation and CREB-dependent gene expression (Ghiani et al., 2007).

**Figure 1:**
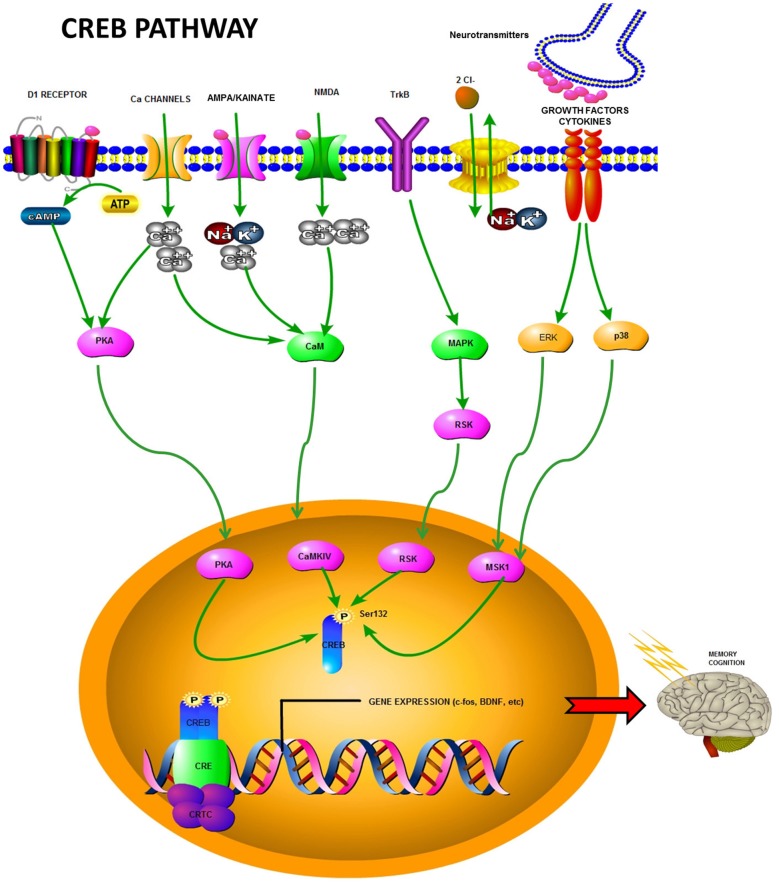
**Summary of the CREB pathway highlighting some of the involved receptors, kinases, and other molecules.** After activation via phosphorylation, CREB induces transcription of different factors such as c-fos and BDNF, some of which are involved in memory and cognition.

The phosphorylation of a serine residue (S133) in KID regulates CREB activity. This phosphorylation establishes an effector role for CREB, initiating the translation of extracellular stimuli into gene expression (Yamashima, 2012). Indeed, CREB phosphorylation facilitates signal transduction, and molecules responsible for its phosphorylation/dephosphorylation are, therefore, highly relevant as modulators of the target gene status. There are a number of signaling cascades upstream of CREB phosphorylation, such as the cAMP pathway (Montminy and Bilezikjian, 1987), and protein kinase pathways (e.g., PKA and CaMKII and IV; Yamashima, 2012). These cascades are activated by stress, inflammatory cytokine activity (MAPK or phosphoinositide 3-kinase (PI3)/Akt pathways; Yamashima, 2012), growth factor/receptor tyrosine kinase activity (Ras/Erk/RSK2 pathway; Ghosh et al., 1994), and phospholipase C (PLC)-PKC signaling (see Figure 1). All these cascades have been shown to induce CREB phosphorylation (Du and Montminy, 1998; Lonze and Ginty, 2002). For example, CREB phosphorylation is facilitated by membrane-bound NMDA receptors (Ghiani et al., 2007), the non-receptor tyrosine protein kinase c-Src (Zhao et al., 2003a), fibroblast growth factor receptor 1 (FGFR1; Hu et al., 2004), and estrogen receptors (Sharma et al., 2007), all of which are part of distinct intracellular cascades. The molecules involved in the modulation of CREB phosphorylation include several neurotransmitters [e.g., dopamine, glutamate, serotonin, gamma-aminobutyric acid (GABA)], growth factors (e.g., insulin-like growth factor 1 IGF-1; vascular endothelial growth factor, VEGF), and neurotrophins (brain-derived neurotrophic factor, BDNF; Yamashima, 2012). A summary of CREB pathways is depicted in Figure 1.

CREB-mediated transcription requires CREB phosphorylation, but the latter is not a reliable predictor of target gene activation; additional regulatory processes are required for the engagement of transcriptional promoter elements. For example, clustering of CBP (CREB-binding protein) has recently been described as crucial, and the CBP paralog p300 enhances CREB functionality. CBP and p300 are both chromatin remodelers (histone acetyltransferases) involved in unwinding promoter chromatin and providing the RNA polymerase II (Pol II) transcription machinery access to promoter DNA (Chan and La Thangue, 2001). If CBP/p300 does not cluster at a gene promoter after CREB phosphorylation, the gene remains transcriptionally silent (Merz et al., 2011). Thus, many co-factors, including some that are directly affected by neuronal activity and growth factors, influence CREB-mediated regulation at the final stage (Merz et al., 2011). This allows CREB signaling to control expression of a variety of target genes.

CREB was first isolated from undifferentiated neuron-like PC12 cells (Montminy and Bilezikjian, 1987) and from the mouse brain (Yamamoto et al., 1988). Interest in CREB’s role in the nervous system has increased in the last few decades, and it has been proposed to be a key regulator of several complex processes ranging from development to plasticity (Yamashima, 2012). For example, CREB has been postulated as a regulator of cell survival, proliferation, and differentiation in the developing brain, whereas its roles in the adult brain include learning, memory, and neuronal plasticity (Yamashima, 2012). It is a key molecule involved in LTM in the majority of species (Bourtchuladze et al., 1994; Yin et al., 1994).

**Figure 2:**
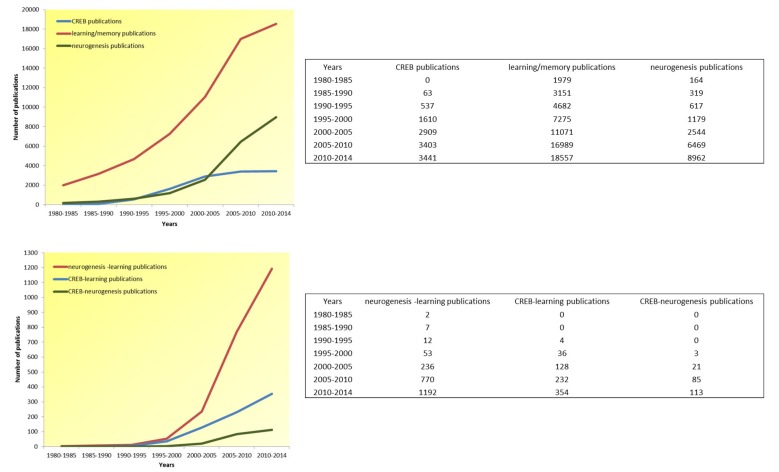
**Article publication trend in the last 34 years (1980–2014).** In over three decades, there has been an exponential increase in the number of publications addressing all topics of interest here (i.e., CREB, learning-memory and neurogenesis), as well as publications addressing the confluence of these interrelated topics (i.e., neurogenesis-learning, CREB-learning, and CREB-neurogenesis), pointing to the scientific recognition of these topic’s mutual relevance.

CREB participation in memory processes has been widely analyzed using animal models ranging from nematodes to higher mammals. The use of different CREB-mutant mice has been one of the most important approaches in elucidating CREB’s role in these memory processes (see review Kida, 2012). CREB has been implicated in the regulation of embryonic and adult neurogenesis (Dworkin et al., 2009). Both neurogenesis and memory, which have been shown to be highly related, constitute an important field of research (see Figure 2). It is widely accepted that adult neurogenesis is involved in memory consolidation and pattern separation, and impairment of these processes has been described in various diseases associated with memory loss, including Alzheimer’s disease (AD; Taupin, 2009; Braun and Jessberger, 2014; Fitzsimons et al., 2014; Cameron and Glover, 2015).

## Current Understanding of CREB, Neurogenesis, and Memory

There were fewer scientific publications on CREB until the 1990s after which the number of publications on this topic have increased. This rise has been accompanied by an exponential increase in publications on learning and memory. Although memory and learning have been studied for a long time, only recent advances in molecular and cellular techniques have enabled researchers to investigate the mechanisms underlying these processes. These advances have permitted the study of complex events such as gene expression in greater detail. Considering that the DNA helix was discovered in 1953, transcription factors (e.g., CREB) are relatively new concepts. Moreover, ideas about neurogenesis have evolved since its discovery 50 years ago (Figure 2).

This review focuses on the most relevant and recent studies on CREB’s role in memory regulation and its participation in neurogenic processes. CREB influences cognitive processes directly by affecting memory and indirectly by affecting adult hippocampal neurogenic capacity. CREB’s affects memory consolidation through its regulation of adult hippocampal neurogenesis (AHN), which mainly occurs in the hippocampal subgranular zone (SGZ) of the dentate gyrus (DG). This mechanism provides a novel perspective on memory consolidation within the adult hippocampus.

## Adult Neurogenesis

The existence of “adult neurogenesis” in mammals was first confirmed 50 years ago (Altman and Das, 1965). Adult neurogenesis refers to the generation of new neurons in the adult brain. Despite its importance, this emergent concept remained obscure until neurogenesis was found to occur in the brain of adult humans (Eriksson et al., 1998). Neurogenesis was traditionally understood to be mainly an embryogenetic phenomenon, but new research has shown the generation of new nerve cells in several areas of the adult brain, including the DG, subventricular zone (SVZ), olfactory bulb (OB), and other areas where it was recently observed (e.g., the cortex and hypothalamus (Gould, 2007). Adult neurogenesis involves cell proliferation, survival, and cell differentiation. AHN, which occurs for specific brain functions, involves not only new neuron formation but also integration of these new-born neurons into functional networks. From this perspective, AHN facilitates memory consolidation via formation of networks (Deng et al., 2010; Weisz and Argibay, 2012). Furthermore, AHN provides plasticity required in memory processes and allows for “pattern separation mechanisms, ” which is crucial for memory consolidation (Bruel-Jungerman et al., 2007; Sahay et al., 2011a; Bekinschtein et al., 2013; Yassa and Reagh, 2013). It is critical to understand this phenomenon because neurogenesis also occurs after brain injury or during brain alteration (e.g., epilepsy); however, in these cases, it is a non-functional process as it is not related with their normal brain function and does not involve wiring of new neurons into networks (Zhang et al., 2014).

### AHN and Memory

In the adult brain, neural stem cells (NSCs) are found in the SGZ, a specific DG subregion (Fanselow and Dong, 2010). These NSCs give rise to new neurons, which are incorporated as new granule cells into hippocampal networks. These networks contribute to learning (Zhao et al., 2003b; Joiner, 2009; Zhang et al., 2009) and memory (van Praag et al., 1999, 2002). In contrast, the ventral DG hippocampus (ventral SGZ) has been implicated in emotion-related disorders (Fanselow and Dong, 2010), including stress, depression, and anxiety. Studies on the types of memories related to AHN have highlighted its role in trace fear conditioning, recognition, spatial memory, pattern separation, and non-mnemonic tasks (reviewed in Cameron and Glover, 2015).

Numerous studies have focused on the relationship between AHN and BDNF. It is widely accepted that BDNF stimulates neurogenesis (Zhang et al., 2011) and is expressed solely in the hippocampus with no additional sources from the peripheral blood. Increased BDNF levels have been shown to enhance hippocampal neurogenic capacity, resulting in improvements in hippocampus-dependent memory (Hsiao et al., 2014). The effects of voluntary exercise and enriched environments on BDNF levels and adult neurogenesis have also been widely studied (Bekinschtein et al., 2011; Yu et al., 2014). IGF-1, another growth factor involved in the BDNF pathway, was also found to affect AHN. Exercise increases IGF-1 levels, which leads to increments in AHN with subsequent improvements in memory and learning. Serotonin and noradrenaline release also upregulate BDNF, explaining the high BDNF levels and neurogenesis following antidepressant treatment (Albert and Benkelfat, 2013).

In summary, adult neurogenesis is considered essential for memory consolidation. Recently, neurogenesis has been discovered to occur in more brain areas; however, memory-related neurogenesis primarily occurs in the DG of the hippocampus and OB. The adult hippocampus has been widely studied for its role in the development of numerous neurodegenerative and neuropsychiatric disorders characterized by decreased AHN. Because hippocampus-dependent memory processes are altered in such diseases, understanding the underlying molecular mechanisms is important for restoring normal brain function. Indeed, some recent drug discovery efforts have focused on increasing AHN.

### AHN and Olfaction

Neurogenesis attributed to the SVZ, the largest neurogenic niche, is also responsible for generating astrocytes, oligodendrocytes, and OB interneurons (Lois and Alvarez-Buylla, 1994); which are involved in “olfactory memory” (enhanced odor differentiation; Sahay et al., 2011b). In fact, a reduction in the generation of olfactory interneurons during adulthood is associated with a decreased ability to discriminate odors (Gheusi et al., 2000). Considering that humans have poor olfactory memory, the role of AHN in olfaction in humans needs to further explored.

## CREB and Adult Hippocampal Neurogenesis

AHN is a highly regulated process, and several transcription factors have been reported to be involved in this regulation (Hodge and Hevner, 2011). In this regard, the role of CREB in AHN process has been widely investigated. The presence of phosphorylated CREB (pCREB) has been found in many new-born immature neurons in the most important neurogenic niches, including the DG in the SGZ (Nakagawa et al., 2002a,b; Jagasia et al., 2009), SVZ, and OB system (Giachino et al., 2005; Herold et al., 2011). CREB activation in the DG resulting in postnatal hippocampal neurogenesis was first reported in Young et al. (1999). Jagasia et al. (2009) found that CREB expression in the DG persists for 3–21 days after cell generation and overlaps with doublecortin (DCX) expression. DCX is a microtubule-associated protein characteristic of immature neurons and precursor cells. The expression of pCREB subsided with the emergence of mature neuronal markers such as calbindin in mature granule cells (Jagasia et al., 2009) or NeuN in the SVZ/OB neurogenic system, where pCREB presence declines along a rostral-to-caudal gradient (Giachino et al., 2005; Herold et al., 2011).

CREB signaling in adult neurogenesis has been widely studied using *in vivo* and *in vitro* approaches. Two groups used rolipram, an inhibitor of phosphodiesterase-4-enhancing CREB signaling, (Nakagawa et al., 2002b; Fujioka et al., 2004) to examine CREB function *in vivo*; they reported an increase in different parameters of adult neurogenesis in the SGZ, including a higher survival rate of new neurons, more precursor cell proliferation, and enhanced neurite outgrowth and dendritic branching (Merz et al., 2011). CREB/CREM signaling has been described as crucial for survival of adult-born neurons. The positive effects of CREB in AHN were inhibited when CREB expression was repressed. In addition, Giachino et al. (2005) reported a significant decrease in number of newborn cells in the OB of transgenic CREM-null knockout mice. Other studies have shown that rolipram’s effects on the morphology of immature and mature neurons in the adult hippocampal DG were eradicated when CREB signaling was repressed by expressing a dominant-negative CREB inhibitor (Fujioka et al., 2004).

The role of CREB signaling in neuronal activation and during survival stages of neurogenesis is better understood than its effects on cell migration and proliferation (Dworkin and Mantamadiotis, 2010). In addition, the pharmacological and genetic approaches used to study the relationship between CREB and AHN do not only affect newborn neurons. Therefore, whether CREB signaling exerts its action by controlling NSCs/new neurons or altering the neurogenic context is an open question warranting further research.

Glucocorticoids are widely considered to negatively influence adult neurogenesis. For example, dexamethasone given at postnatal day 6, a crucial point in the generation of hippocampal neural precursors that survive in the adult brain (Ortega-Martinez, 2015; Ortega-Martínez and Trejo, 2015), suppresses AHN, decreases cell proliferation, and induces behavioral impairments (e.g., learning deficits and increased anxiety; Ortega-Martinez, 2015; Ortega-Martínez and Trejo, 2015). Notably, increased levels of corticosterone, which is known to decrease neural precursor cell proliferation and contribute to neuronal atrophy, were also correlated with lower pCREB levels (Yu et al., 2004).

Various studies have demonstrated that inhibition of CREB signaling with the dominant-negative CREB inhibitor A-CREB (Ahn et al., 1998; Herold et al., 2011) significantly decreases newborn cell survival (Jagasia et al., 2009). New cells showed loss of expression of DCX and Pax6 transcription factor (both of which are related to cell maturity), impairments in rostral migratory stream (RMS) migration, decreased cell proliferation and survival, and increased morphological defects in newborn OB cells (Herold et al., 2011; Merz et al., 2011). The effects of CREB-signaling suppression show that this pathway is crucial for proliferation, differentiation, survival, maturation, and functionality in adult neurogenesis sites (mainly the OB, SVZ, and DG; Merz et al., 2011).

These assumptions are also pertinent to *in vivo* experiments that have described how CREB signaling affects proliferation of NSC precursors enhancing this cellular mechanism (Kim et al., 2002; Dworkin et al., 2009; Grimm et al., 2009; Merz et al., 2011).

The epigenetic mechanisms of DNA methylation has also been shown to regulate CREB signaling. The CRE sequence contains a CpG island that can be methylated; methylation inhibits binding and restricts CREB localization to functional sites (Zhang et al., 2005). Furthermore, neuronal activity regulates DNA methylation via different enzymes such as DNA-demethylase Gadd5b, suggesting a new link between CREB signaling and neuronal activity, which may be important in adult neurogenesis (Ma et al., 2009).

Another mechanism by which CREB can regulate neurogenesis is by affecting crucial microRNAs (s). miRNAs are ncRNAs (non-coding RNAs) that generally contain 19–23 nucleotides and regulate translation through one of two mechanisms: degradation of target mRNAs or inhibition of the translation machinery (Merz et al., 2011). miRNAs are known to control both precursor cell survival and differentiation in adult neurogenesis. Notably, CREB controls the expression of both MeCP2 (Klein et al., 2007; Chahrour et al., 2008). miRNA-132, which is a member of a cluster of miRNAs enhanced by neuronal activity (Fiore et al., 2009), participates in neuronal morphogenesis, and regulates cognitive capacity (Vo et al., 2005; Hansen et al., 2013). miRNA-134 regulates CREB post-transcriptionally (Gao et al., 2010). In addition, factors known to regulate the proliferation of neural precursor cells (e.g., Wnt; Hirsch et al., 2007; Dworkin and Mantamadiotis, 2010) have been shown to enhance CREB signaling (Chen et al., 2005) in cultured cortical neurons (Vo et al., 2005).

All of the factors described above activate or inhibit CREB signaling, with or without affecting gene expression. Among the molecules related to adult neurogenesis that are also affected by CREB signaling, it is important to highlight the following: BDNF (Kida, 2012; Sable et al., 2014), prolactin (Wang et al., 2013), bcl-2 (Fujii et al., 2014), polysialylated neuronal cell adhesion molecule (PSA-NCAM; Park et al., 2004; NGF; Lim et al., 2014), and cyclin D2 (Kowalczyk et al., 2004; White et al., 2006).

One hypothesis regarding the influence of CREB on adult neurogenesis comes from a recent study that focused on polyunsaturated fatty acid (PUFA)-GPR40-CREB signaling (Yamashima, 2012). The authors showed that primate neurons possess a PUFA-GPR40-CREB-signaling pathway that exerts its action in adult newborn neurons by enhancing the synthesis of BDNF and PSA-NCAM, both of which are implicated in adult neurogenesis (Yamashima, 2012).

A recent review discussed the functional roles of CREB as a positive regulator in the formation and enhancement of memory (Kida and Serita, 2014). Different genetic models used for the study of CREB function were analyzed. Interestingly, some of the findings are in accordance with the new perspective of CREB modulation of memory via its role in adult neurogenesis. In this regard, several transgenic lines of CREB mice such as CREB-VP16, Y134F and DIEDML, which show enhanced STM, also exhibit upregulation of hippocampal BDNF expression, which is related to higher AHN. In addition, microinfusions of BDNF or a BDNF inhibitor into the dorsal hippocampus were associated with improved or impaired STM, respectively. These findings indicate new roles for CREB in learning and memory; CREB plays a regulatory role in STM via the regulation of BDNF expression (Suzuki et al., 2011; Kida and Serita, 2014). Another similar study, Lee et al. (2013) showed that phytoceramide administration enhanced memory via the upregulation of hippocampal pCREB and BDNF expression, which resulted in increased AHN. These results suggest that phytoceramide contributes to memory enhancement and increased BDNF expression, which could lead to increased neurogenesis (Lee et al., 2013).

Finally, links between CREB signaling and neuropsychiatric diseases (e.g., depression) have been reported, and there is evidence that subjects with these diseases also have impairments in AHN (Gass and Riva, 2007). Chronic antidepressant treatment can upregulate hippocampal CREB activity, similar to CREB pathway upregulation in AHN. One study reported that CREB-deficient mice with an antidepressive phenotype also showed neurogenesis enhancement. Compared to wild-type mice, these mice responded more quickly to depression treatment.

## CREB and Memory

One emergent hypothesis regarding the prevention of consolidation of STM into LTM involves the inhibition of proteins or transcription factors. This hypothesis also draws on the relationship between memory consolidation and affective and emotional depression-related circuits (Vogt et al., 2014). The translation of proteins from genes is crucial for memory consolidation (Flexner et al., 1965; Alberini and Kandel, 2015). Different pharmacological approaches have been used to block mRNA and protein synthesis, but this results in an impairment in LTM consolidation without affecting STM (Suzuki et al., 2004; Duvarci et al., 2008; Alberini and Kandel, 2015).

Brunelli et al. (1976) found that serotonin increases the level of cAMP in sensory neurons. It was one of the first reports of an association between cAMP and learning and memory (Brunelli et al., 1976). A decade later, Marc Montminy and L.M. Bilezikjian described, for first time, CREB as a cellular transcription factor that binds the CRE– thereby increasing the transcription of the somatostatin gene (Montminy and Bilezikjian, 1987). The first report of a direct role of CREB transcription factors, downstream of the cAMP pathway, in memory-related synaptic plasticity was in Dash et al. (1990). Dash et al. (1990) demonstrated that, during LTM in Aplysia neurons, PKA activates gene expression via CREB. They could selectively eliminate the long-term process by blocking the binding of CREB1 to its DNA response element. The approach used by Dash et al. (1990) was the microinjection of CRE oligonucleotides into sensory neurons co-cultured with motor neurons. These oligonucleotide bound to the CREB1 protein within the cell and inhibited CREB1. In addition, these oligonucleotide prevented CREB1 from binding to CRE sites in the regulatory regions of cAMP-responsive genes, thus blocking subsequent gene expression. The injection of this oligonucleotide selectively blocked LTM, but did not have an effect on the STM.

A number of more recent studies have demonstrated that CREB is the main element underlying the conversion of STM to LTM (Barco et al., 2003; Kim et al., 2013; Vogt et al., 2014). Several mutant mice phenotypes have been developed to investigate the roles of CREB in learning and memory formation. Walton et al. (1992) study employed a learning and memory task in which mice with a tetracycline-controlled transactivator/operator system specifically expressed a dominant-negative inhibitor of CREB activation in CaMKIIα-positive forebrain cells. It was shown that LTM but not STM was impaired. However, these mutant mice did not exhibit a contextual fear-conditioning deficit (Pittenger et al., 2002). In addition, similar results showing that the genetic loss of CREB function impaired LTM but not STM formation has been obtained by other researchers (Bourtchuladze et al., 1994; Kida et al., 2002; Kida and Serita, 2014). Kida (2012) revealed the crucial role of CREB in the consolidation of contextual fear conditioning using tamoxifen-inducible expression of a dominant-negative CREB repressor (Vogt et al., 2014). Furthermore, mutant mice with inhibited CREB activity exhibited deficits in hippocampal late long-term potentiation (L-LTP). Collectively, this evidence indicates that CREB has a central role in these processes and is necessary for both memory consolidation and LTP.

CREB exerts its action as a molecular switch for memory formation (Suzuki et al., 2011). Viral vectors-mediated gene transfer have been widely used to overexpress CREB. Research has shown that the upregulation of CREB activity promotes memory consolidation (Josselyn et al., 2001; Zhou et al., 2009; Alberini and Kandel, 2015). Even from the traditional point of view that CREB enhances LTM, it is thought that the upregulation of CREB-mediated transcription improves STM (Suzuki et al., 2011). It is established that LTM promotion occurs due to enhanced memory consolidation via upregulation of CREB transcriptional activity (Suzuki et al., 2011). In contrast, an increase in basal levels of BDNF, a CREB target gene, is related to improvements in STM. The upregulation of BDNF and CREB activity synergistically improves LTM formation (Suzuki et al., 2011). Therefore, CREB positively controls memory consolidation and is involved in controlling BDNF expression, which also affects memory processes (Suzuki et al., 2011) and is necessary for AHN.

The main CREB target-gene expression related to memory consolidation and LTP are c-fos, activity-regulated cytoskeleton-associated protein (Arc), and BDNF (Miyamoto, 2006; Alberini and Kandel, 2015). Improvements in memory consolidation have also been attributed to overexpression of CREB or Y134F in the amygdala and hippocampus (Josselyn et al., 2001; Restivo et al., 2009; Sekeres et al., 2010). Studies that used viruses to overexpress CREB in the lateral amygdala, which is involved in the fear memory network (Han et al., 2007; Giachero et al., 2015), demonstrated that neurons in this brain area are functionally interrelated (Han et al., 2007). These neurons (i.e., those overexpressing CREB in the lateral amygdala) exhibit greater neuronal excitability (Zhou et al., 2009). These findings indicate that neurons with high levels of CREB activation may contribute to improved memory via involvement in the memory network (Kida and Serita, 2014).

In addition, some neuropsychiatric or neurodegenerative diseases such as depression (Zaninotto et al., 2015), schizophrenia (Burton and Twamley, 2015), and AD (Takeda et al., 2014) are associated with memory loss. In this regard, CREB has been postulated to change the sensitivity of the nucleus accumbens to rewarding and aversive drugs (Bilbao et al., 2014a,b). Because hippocampal CREB expression is upregulated by chronic antidepressant treatment, CREB activity appears to be involved in the pathogenesis and treatment of depression (Gass and Riva, 2007).

Taken together, these studies demonstrate that CREB plays a key role in the formation, consolidation, and enhancement of both STM and LTM. Importantly, in addition to its direct effect in memory processes, CREB may influence AHN directly or through its ability to modulate gene expression. That is, CREB modulates AHN, which affects memory consolidation. CREB mainly contributes to AHN by affecting the expression of genes such as BDNF or PSA-NCAM, and also directly participates in neurogenesis via its signaling in newborn neurons. Moreover, CREB signaling pathways are a target of drug therapies to ameliorate brain disorders associated with cognitive dysfunction (e.g., learning and memory impairments; Kida and Serita, 2014), and some of these drugs are designed to improve memory by stimulating AHN.

## Concluding Remarks Regarding the Implications for CREB’s Role in Memory via AHN Regulation

**Figure 3:**
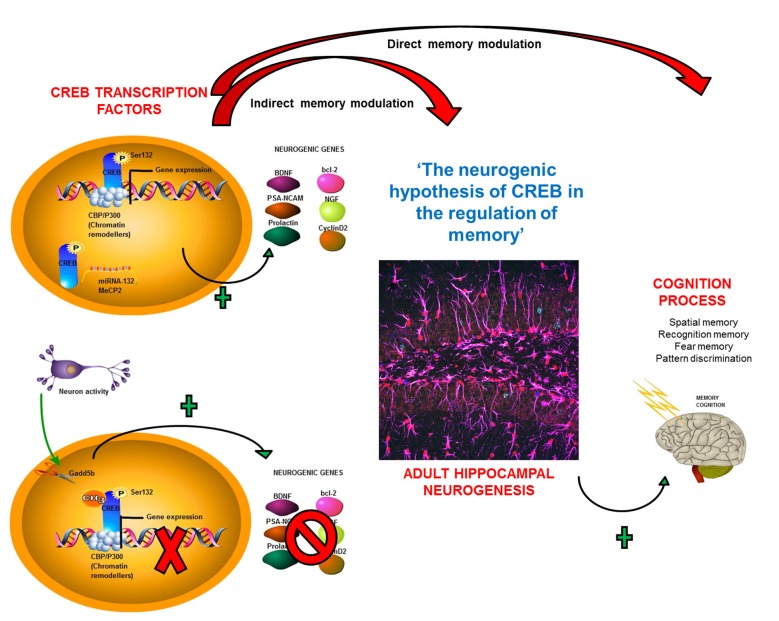
**Neurogenic hypothesis for CREB’s role in the regulation of memory.** A summary of how CREB can influence this cognitive process of memory formation through its direct participation in, or through its modulation of, adult hippocampal neurogenesis (AHN), constituting a new theory for CREB regulation of memory.

This review has examined how CREB regulates both adult neurogenesis and memory. With regard to the former, enhanced CREB signaling has been widely described to promote newborn neuron survival, precursor cell proliferation, and neurite outgrowth, and dendritic branching (Giachino et al., 2005; Merz et al., 2011). CREB regulation has multiple aspects, including its phosphorylation and recruitment of chromatin remodelers such as CBP and p300, which are essential for CREB gene transcription (Merz et al., 2011) This process is also regulated by multiple cofactors (Merz et al., 2011) facilitating the transcription of different genes involved in AHN mechanisms, such as the expression of BDNF, PSA-NCAM, and NGF2 (Figure 3). Furthermore, CREB regulates AHN directly by affecting gene expression and indirectly by influencing epigenetic mechanisms such as inhibitory DNA methylation in its CpG island (Zhang et al., 2005). Neuronal activity controlling DNA methylation patterns (Ma et al., 2009) establish interdependence between these two integrated processes (i.e., neuronal activity and CREB activation). Another mechanism by which CREB can control AHN is by mediating miRNA expression (Vo et al., 2005; Klein et al., 2007; Chahrour et al., 2008; Figure 3).

The relationship between CREB and memory consolidation has been widely studied since Brunelli et al. (1976). It is now accepted that CREB is the key molecule responsible for converting STM to LTM and that it plays a central role in memory consolidation (Barco et al., 2003; Kim et al., 2013; Vogt et al., 2014). It has also been established that CREB mainly affects STM by enhancing BDNF gene expression (Suzuki et al., 2011). Finally, hippocampal CREB overexpression improves memory formation (Restivo et al., 2009; Sekeres et al., 2010).

Taken together, the existing literature suggests that CREB enhances memory through several different mechanisms. Directly, it can increase expression of genes that modulate memory. The new perspective discussed here is that CREB modulates AHN, which also affect memory processes. In this regard, CREB enhances the expression of target genes such as BDNF and PSA-NCAM, which augment AHN. In addition, CREB upregulation itself promotes AHN, leading to greater neuronal survival and postnatal hippocampal neurogenesis (Cameron and Glover, 2015). CREB was also recently shown to be crucial for AHN. AHN has a central role in several cognitive processes including spatial, recognition, and fear conditioning memory (Cameron and Glover, 2015; Figure 3). Based on the evidence, it is reasonable to formulate the novel hypothesis referred to here as “A new perspective of memory regulation based on CREB-mediated hippocampal neurogenesis” in which CREB controls memory processes by direct regulation and effects on adult neurogenesis, which affects cognitive and memory processes.

In addition, it is important to not underscore that cognitive process attributable to CREB transcription factors are complex and difficult to study. In this regard, evidence for this hypothesis is crucial to design follow-up studies. Even though there is considerable accumulated knowledge in this domain, it is necessary to better understand how CREB works in adult neurogenesis. Many questions remain unanswered. For example, can CREB also modulate the neurogenic niche or directly affect new neurons? It would be interesting to clarify whether CREB can modify the SGZ microenvironment to facilitate AHN. Another important question is whether CREB can modulate or change the phenotype of a specific neural lineage (e.g., stem or progenitor cells instead of mature neurons). Appropriate gene expression is crucial in determining phenotype, and the transcription of different genes affects diverse mechanisms including cell quiescence, proliferation, and death. Finally, can CREB modulate adult neurogenesis through the control of other adult neurogenesis regulators such as neurotransmitters, steroid hormones, or cytokines (Ortega-Martinez, 2015)? Importantly, due to its effect on AHN, CREB may be a strategic target in the development of therapeutic drugs for neurodegenerative and psychiatric diseases associated with cognitive impairment.

## Author Contributions

SO-M is the sole author of this paper and is responsible for its content.

## Conflict of Interest Statement

The author declares that the research was conducted in the absence of any commercial or financial relationships that could be construed as a potential conflict of interest.

## Abbreviations

CREB: cAMP-responsive element-binding protein
AHN: adult hippocampal neurogenesis
DG: dentate gyrus
SGZ: subgranular zone
SVZ: subventricular zone
KID: central kinase-inducible domain.
